# Dermatology

**Published:** 1908-06

**Authors:** J. A. Nixon


### DERMATOLOGY.

The study of **tuberculosis of the skin** has attracted so much attention of recent years, particularly since Finsen's therapeutic researches, that a complete review of the present state of our knowledge of the subject is very apposite. An excellent summary has been made by Alexander, of Berlin, which helps materially to a proper appreciation of the vastly increased importance now allotted to tuberculosis as a factor in skin diseases.

In **diagnosis** he draws attention to the value of tuberculin injections, which have been so profitably employed by Neisser, Jadassohn and others to demonstrate the tuberculous nature of various dermatoses. The method is of course familiar, consisting in the subcutaneous injection of a small quantity of Koch's "alt-tuberculin" into a suitable spot (dose, ½–1–5–10 milligrammes), when the appearance of a local reaction and a rise of temperature is found to result in tuberculous subjects. This reaction to tuberculin has been found to be most constant in lupus vulgaris, but it is only a little less certain in many other forms of tuberculosis cutis, except in the verrucous variety (verruca necrogenica), when for some reason, possibly the presence of so much fibrous tissue, the reaction is often absent.

More important, however, than the fact that occasionally there is a negative result in a tuberculous case, is the knowledge that no other cutaneous affection, especially tertiary syphilis, which presents in practice the greatest difficulties as regards differential diagnosis, will react to tuberculin.[1]

The only possible exception is tubercular leprosy, which gives an apparently similar reaction, but the temperature as a rule rises later or repeatedly, whilst the accompanying reaction shows characteristic variations.

A *general* as well as *local* reaction may be relied upon with almost absolute certainty for lupus, and in most instances a well-marked *local* reaction alone may be regarded as equally dependable. On the other hand, the *general* reaction without the *local* does not answer the question as to the skin condition, for there may be some other tuberculous focus in the body causing the temperature to rise with the injection. The absence of the *general* reaction may be interpreted as a probability of the non-tuberculous nature of the dermatosis, but Jadassohn has pointed out that this is not conclusive, for possibly very small cutaneous foci will not suffice to disturb the temperature.

Alexander insists on the necessity for exact technique in making the injection. The solution must be fresh. A series of injections must be made starting with $\frac{1}{2}$ mg.; if no reaction follows, a second injection of 1 mg. is given after two days' interval, and so on if necessary to 5 mg., and finally to 10 mg. This "progressive" dosage must be strictly adhered to if the results are to be trustworthy. In children the dose should increase from $\frac{1}{20}$—$\frac{1}{10}$ mg. up to 1—3 mg., and in older children even to 5 mg. The newer forms of tuberculin, although useful for treatment, are unreliable for diagnosis.

In cases complicated with pulmonary tuberculosis greater caution has to be exercised, both as regards the size of the dose (a trial injection of $\frac{1}{100}$—$\frac{1}{10}$ mg. must be given) and as to the interpretation of the results.

The **clinical manifestations** of tuberculosis of the skin have recently been more thoroughly investigated.

*Lupus vulgaris* is now generally recognised to commence in about seventy-five per cent. of cases in the mucous membrane of the nose, and to spread to the skin secondarily, so that most cases of lupus of the face should perhaps come in the first instance under the observation of the rhinologist.

[1] Alexander, *Berl. klin. Wchnschr.*, 1907, xliv. 314, 344, 377.

Holländer distinguishes two varieties of lupus of the face, that affecting primarily the skin (cheeks, &c.), growing slowly, and not causing great destruction of tissue ; and that affecting primarily the nasal mucous membrane, the more common variety, spreading rapidly, eroding the nose and attacking before long the face itself. "Lupus centralis faciei," Holländer has called it, and he pleads for a very active treatment particularly directed to ensuring freer nasal respiration (even by operation) with phototherapy.

Holländer also advises abortion if tuberculous foci are detected during pregnancy.

*Lupus carcinoma,*[1] that is to say carcinoma starting either in an active patch or an old scar of lupus, is a condition only lately described ; in 1901 Ashihara collected accounts of 126 cases. The pertinent question is raised whether the X-ray treatment may not be responsible for this hitherto unknown tendency in lupus. "The possibility of such a connection should always be borne in mind, for it is well known that chronic X-ray dermatitis gives rise occasionally to cancer of the skin."

The possibility of tuberculosis cutis assuming malignant forms has been more than once suggested, and there is no doubt now that in particular situations, *e.g.* the alæ nasi and lobule of the ear, simple tuberculides may display a tendency to tumour-formation. Henk has found recorded fourteen cases of these "tuberculous tumours," which in their clinical course can only be described as "malignant."

*Lupus Pernio* is now an accepted fact (we notice that Hutchinson's long struggle on its behalf goes unrecognised in this paper of Alexander's), and it is admitted that the defective circulation which gives rise to chilblains also lays open the affected parts to invasion by the tubercle bacillus.

*Lupus follicularis disseminatus faciei* (of Tilbury Fox) appears as an acute or sub-acute eruption confined to the face in the shape of minute brownish-red or livid spots, soft, slightly raised, the size of a pin's head, sometimes apparently forming in the follicles ; it must not be confused with the diffuse lupus which occasionally follows measles (*lupus disseminatus post-exanthematicus*), nor the very rare *generalised miliary tuberculosis* involving the skin with other parts.

*Verruca necrogenica* is referred to as showing a partiality for the Westphalian coal miners, possibly, as Fabry suggests, because under the influence of coal dust wounds remain open, yet aseptic, so that the tubercle bacillus finds itself in undisputed possession of the inviting surface.

Another group of skin affections less certainly tuberculous are classed under the term :—

[1] See also Sequeira, *Brit. J. Dermat.*, 1908, xx. 40.

*Tuberculides.* These include—

| | |
|---|---|
| Folliclis. | Lupus erythematosus. |
| Lichen scrofulosorum. | Chilblain. |
| Erythema induratum. | Angeiokeratoma (Mibelli). |

The distinction between a *tuberculide* and *tuberculosis cutis* may be said to be that while the latter is a local affection, depending on the presence of the tubercle bacillus, the former is in some way the product of the bacterial metabolism, so that Hallopean has even suggested the portmanteau word " toxituberculide " as more fully expressive of our present views on the nature of tuberculides.

But it is worth noting that Jacobi and Wolff have demonstrated the tubercle bacillus in the eruption of lichen scrofulosorum, and Pellizari and Haushalter obtained positive results from inoculation of animals ; that Philippson and Macleod have discovered bacilli present in folliclis, and that Thibièrge and Ravaut, Colcott Fox and Carle-Lyon have proved that erythema induratum is pathogenic for animals. Moreover, many of these tuberculides will give a positive tuberculin reaction. So we may conclude that at least some part of the tuberculide contains a living virus capable of propagation.

**Treatment** is dealt with very fully, particularly in the case of lupus, where the following general methods are in vogue.

*Surgical* (excision, scraping, &c.).—This is the most certain and effectual means of eradicating lupus, and should always be resorted to in a situation where the scar is of no moment. The skin should be excised over the area of surrounding skin which shows any sign of the local tuberculin reaction (Scholtze, Buschke, &c.), or speaking roughly, a margin of ½ to 1 cm. beyond the visible edge of the disease should be removed.

The hot air treatment of Holländer is especially valuable for tuberculosis of mucous membranes (nasal lupus) or for localised cutaneous foci. In most cases this treatment is not applied continuously, but is used for a short time as a preliminary to some other milder method, *e.g.* Finsen light.

Paquelin's cautery is recommended for small solitary nodules of lupus.

Among *chemical methods* Unna's stabbing with pure carbolic is excellent for circumscribed patches in which the nodules are fairly discrete. Arsenical and antimonial pastes are still applicable, and a newer corrosive, which displays an elective affinity for lupus in preference to normal skin, is Pyrogallol, first suggested in 1879 by Von Jaksch. Pyrogallol vaseline (10 per cent.) applied to the affected part for three or five days, followed by some simple emollient ointment, is said to prove successful even in the most refractory cases of verruca necrogenica. Ehrmann suggests 33 per cent. resorcin ointment in place of the foregoing, and combines with it X-ray exposures, while other dermatologists employ for the same purpose crude hydrochloric acid saturated with chlorine.

*Phototherapy* includes Finsen's light (with its many modifications in the way of "iron-carbon points," Kromayer's quicksilvered quartz-lamp), radium, and the X-rays. Alexander regards the results as too well known and established to require very detailed consideration.

Lastly, he refers to the therapeutic use of tuberculin injections which Lassar, Blaschko and Lesser claim to have seen produce such good results in tuberculosis of the skin.

Alexander does not claim for any method an absolute superiority; he pleads for the healing of the patient, not a treating of the disease, and suggests the alternation and combination of any and every remedy, internal or external, which the behaviour of the local lesion and the general well-being of the patient may warrant.

J. A. Nixon.

